# Perforated appendicitis presenting as mechanical bowel obstruction in a pediatric patient: a rare diagnostic challenge

**DOI:** 10.1093/jscr/rjaf628

**Published:** 2025-08-13

**Authors:** Mohammed Al Blooshi, Sadaf Binu Manaf, Munir Ahmad, Mamoun Al Marzouqi

**Affiliations:** Department of Pediatric Surgery and Urology, Al Jalila Children’s Specialty Hospital, 6th Street, Al Jaddaf, Dubai 300100, United Arab Emirates; Department of General Pediatrics, Al Jalila Children’s Specialty Hospital, 6th Street, Al Jaddaf, Dubai 300100, Emirate of Dubai, United Arab Emirates; Department of Pediatric Surgery and Urology, Al Jalila Children’s Specialty Hospital, 6th Street, Al Jaddaf, Dubai 300100, United Arab Emirates; Department of Pediatric Surgery and Urology, Al Jalila Children’s Specialty Hospital, 6th Street, Al Jaddaf, Dubai 300100, United Arab Emirates

**Keywords:** perforated appendicitis, small-bowel obstruction, pediatric, MRI, mechanical obstruction

## Abstract

Perforated appendicitis causing mechanical small-bowel obstruction is rare in pediatric patients and can mimic common gastrointestinal illnesses, delaying definitive treatment. We report the case of a 3-year-old girl with 5 days of nonbilious vomiting, diarrhea, and high-grade fever, initially managed as gastroenteritis. Ongoing abdominal distension and failed conservative management prompted plain radiographs showing dilated small-bowel loops and air–fluid levels. Subsequent magnetic resonance imaging revealed a periappendiceal abscess compressing the distal ileum at a transition point. Urgent laparoscopy, converted to a minilaparotomy, confirmed a perforated appendix with dense adhesions tethering the ileum, necessitating appendectomy, adhesiolysis, and peritoneal lavage. The patient’s postoperative recovery was uneventful, with normalization of inflammatory markers and restoration of bowel function. This case highlights the importance of considering atypical appendicitis in prolonged gastrointestinal symptoms, the utility of magnetic resonance imaging in diagnosis, and the need for prompt surgical intervention to prevent serious complications.

## Introduction

Acute appendicitis remains a leading cause of surgical emergencies, accounting for a substantial proportion of acute abdominal conditions in both adults and children [1]. Despite its high incidence, atypical presentations in pediatrics—where nonspecific symptoms often overlap with common childhood illnesses [2, 3]—can delay diagnosis and management. Such delays raise the risk of complications, including perforation, abscess formation, and generalized peritonitis, all of which significantly increase morbidity [4].

Mechanical bowel obstruction secondary to acute appendicitis is exceptionally rare, representing a small fraction of pediatric small-bowel obstruction (SBO) cases [5]. Perforation can produce inflammatory exudates and adhesions that kink or entrap bowel loops, presenting an obstructive picture [6]. Overlapping clinical features with infectious gastroenteritis may lead to misdiagnosis or delayed treatment, placing children at risk of severe outcomes such as bowel ischemia or sepsis [3].

While computed tomography (CT) has improved the detection of SBO, identifying appendicitis-related obstruction preoperatively remains difficult [2]. Pediatric imaging must minimize radiation, and contrast allergies may further limit diagnostic options. In these situations, magnetic resonance imaging (MRI) and focused ultrasound are pivotal, although dilated loops can hinder visualization [5].

We describe the case of a 3-year-old who initially presented with features suggestive of infectious gastroenteritis but was ultimately diagnosed with perforated appendicitis causing acute SBO. This rare presentation underscores the necessity of a high index of suspicion for appendicitis in pediatric bowel obstruction and the importance of timely surgical intervention to prevent life-threatening sequelae.

## Case report

A previously healthy 3-year-old female presented with 5 days of nonbilious, nonbloody vomiting and diarrhea, followed by 4 days of high-grade fever (up to 40°C) and poor oral intake. Initial symptoms included watery stools two to three times daily and cyclical fevers responsive to antipyretics, with mild lower abdominal discomfort but no urinary complaints. Her mother and aunt experienced similar gastrointestinal symptoms after a shared cafeteria meal, prompting an initial diagnosis of viral gastroenteritis. Past medical history was notable only for eczema.

On examination, she appeared pale, tachycardic, and mildly distressed. There was persistent tachycardia despite fluid resuscitation, but normal blood pressure and oxygen saturation. Abdominal evaluation showed mild distension without peritoneal signs. Laboratory results revealed leukocytosis (WBC 14.8 × 10^9^/l, 82% neutrophils), elevated C-reactive protein (288.9 mg/l), and procalcitonin (13.6 ng/ml), alongside mild hyponatremia (Na^+^ 133 mmol/l) and metabolic acidosis ($\text{HCO}^{-}_{3}$ 15 mmol/l). Urinalysis showed trace leukocyte esterase and ketonuria without bacteriuria. Blood, stool, and urine cultures were sterile; peritoneal fluid later grew extended-spectrum β-lactamase (ESBL)-producing *Escherichia coli*.

**Figure 1:**
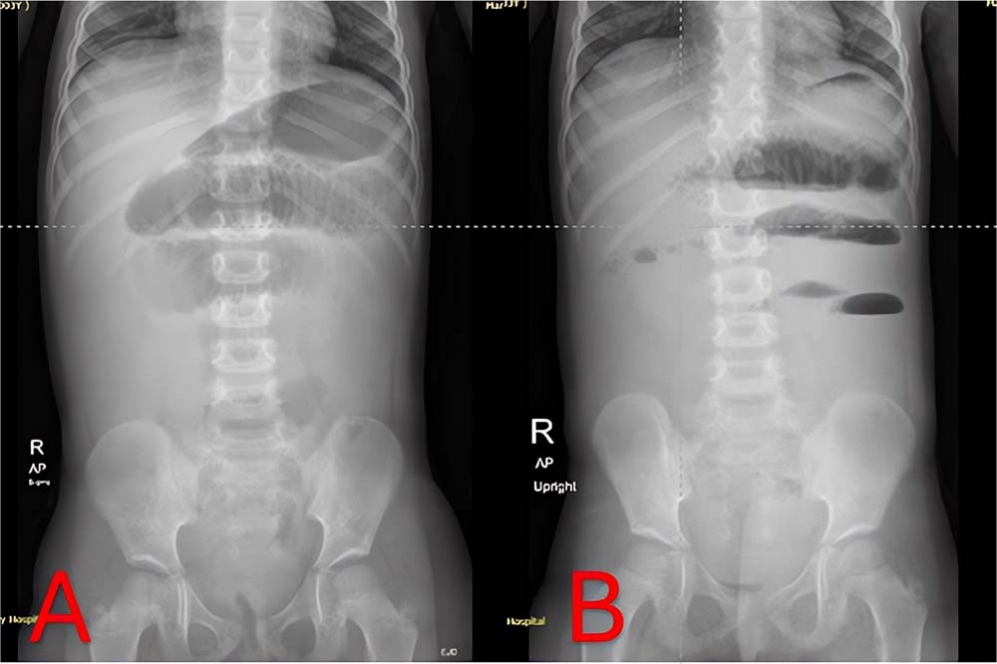
Admission abdominal radiographs (composite). (A) Supine abdominal X-ray on presentation demonstrating central small-bowel dilation and paucity of distal gas. (B) Upright abdominal X-ray on presentation showing multiple small-bowel air-fluid levels without free air.

**Figure 2:**
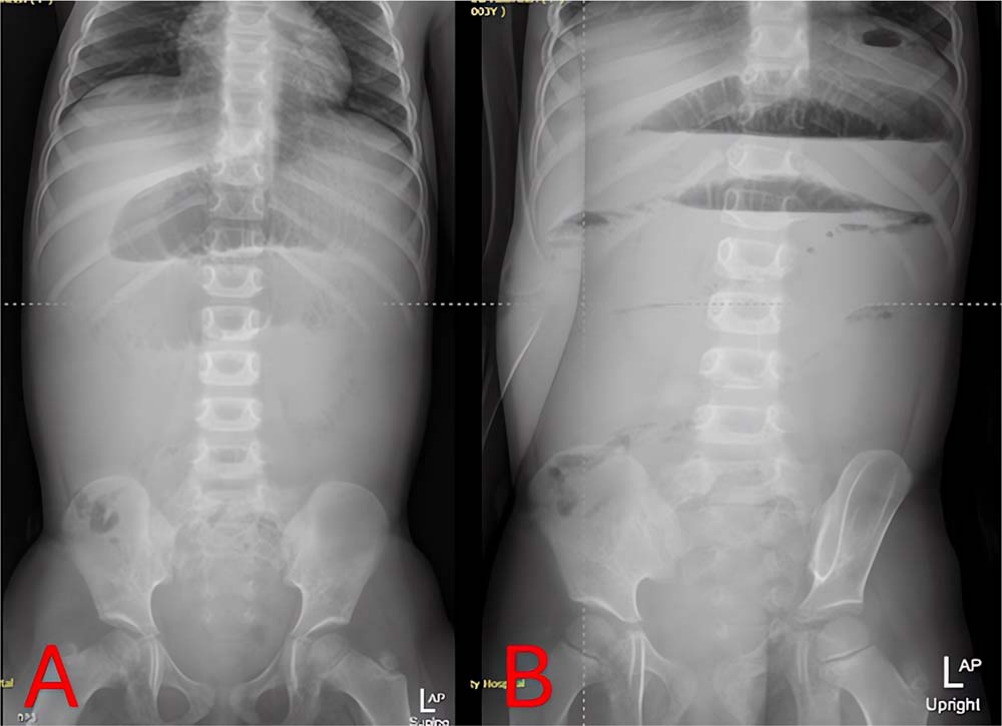
Twenty-four-hour follow-up abdominal X-rays. (A) Supine view 24 hours postadmission with persistent small-bowel distension. (B) Upright view 24 hours postadmission confirming ongoing air–fluid levels and no colonic gas.

Plain abdominal radiographs revealed centrally dilated small-bowel loops with paucity of distal gas (Fig. 1A) and multiple air–fluid levels without free subdiaphragmatic air (Fig. 1B), consistent with mechanical small-bowel obstruction. Initial management, including nasogastric decompression, nil per os (NPO) status, and intravenous ceftriaxone (75 mg/kg once daily) plus vancomycin (15 mg/kg every 6 hours), failed to improve radiographic findings after 24 hours (Fig. 2A and B), prompting further imaging.

**Figure 3:**
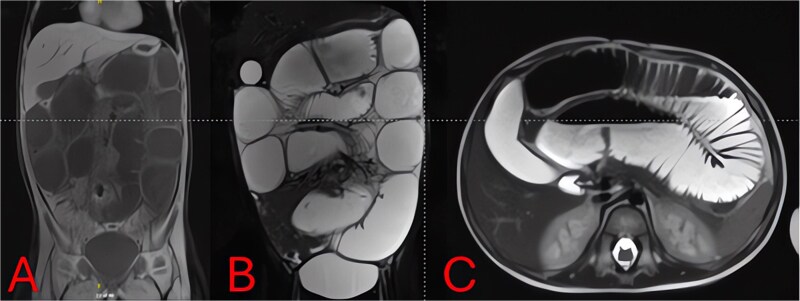
MRI of the abdomen—identification of obstruction cause. (A) Coronal T2-weighted MRI showing multiple fluid-filled, dilated small-bowel loops and a focal right lower quadrant transition point. (B) Axial T2-weighted MRI demonstrating a periappendiceal abscess with mesenteric edema compressing adjacent ileal loops. (C) Sagittal MRI view highlighting appendiceal abscess boundaries and a possible appendicolith.

On hospital Day 2, an abdominal MRI without contrast confirmed marked fluid-filled small-bowel loop dilation and a focal transition point in the right lower quadrant (Fig. 3A). Axial sequences demonstrated a periappendiceal fluid collection with mesenteric edema and a possible appendicolith (Fig. 3B). A sagittal plane showed the abscess tracking into the pelvis (Fig. 3C), indicating perforated appendicitis causing adhesive small-bowel obstruction.

**Figure 4:**
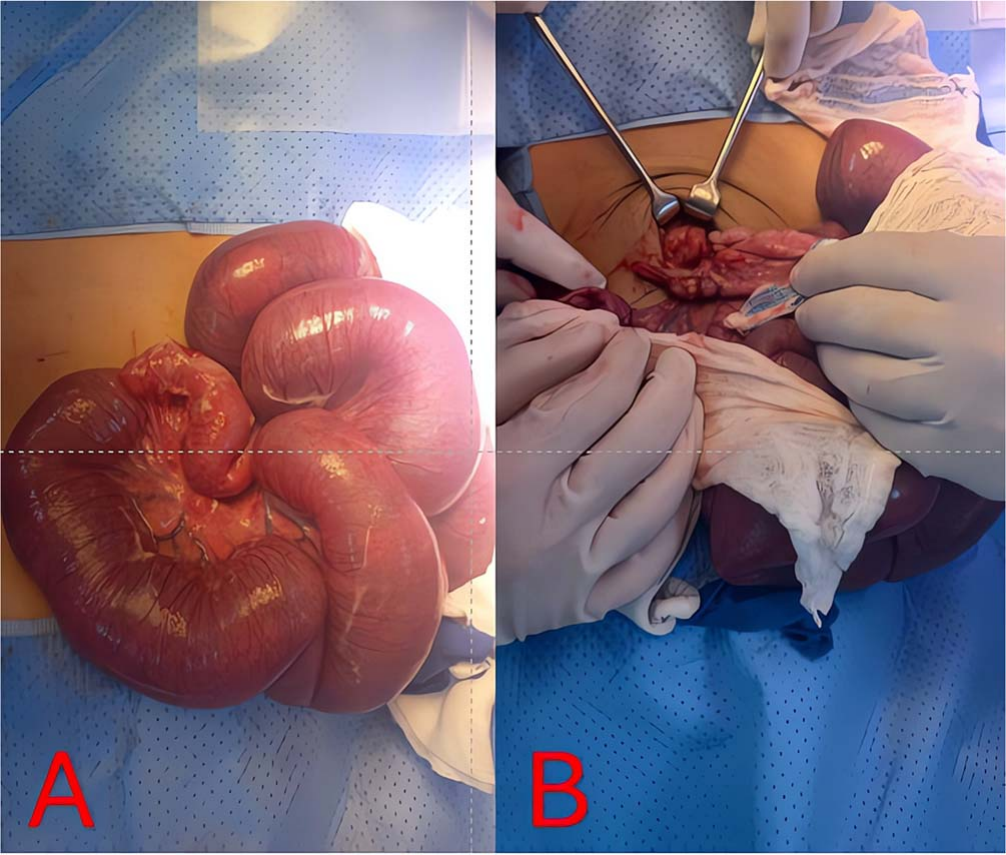
Intraoperative findings and resolution. (A) Intraoperative photo of markedly distended, congested small-bowel loops exteriorized at laparotomy. (B) Intraoperative view of the perforated appendix and surrounding abscess/adhesions tethering the distal ileum, causing mechanical obstruction.

Urgent diagnostic laparoscopy was converted to a minilaparotomy for better visualization. Markedly distended loops were exteriorized (Fig. 4A). Dense inflammatory adhesions tethered the distal ileum to a perforated appendix with a fecolith, creating a fixed kink. Appendectomy with base transfixation, adhesiolysis, repair of a small jejunal serosal tear, and peritoneal lavage were performed (Fig. 4B). A Foley catheter and nasogastric tube were left *in situ* for 24 and 72 hours, respectively.

Postoperatively, she received intravenous meropenem (20 mg/kg every 8 hours for 7 days) followed by 5 days of gentamicin per culture sensitivities. She remained NPO until postoperative Day 3, then progressed her diet without complication. The Foley catheter was removed on Day 2. C-reactive protein (CRP) declined from 288.9 mg/l preoperatively to 13.7 mg/l by Day 7 and 4.1 mg/l by Day 10. A Day-7 ultrasound showed no residual collections. The patient had no wound infection or ileus, tolerated full feeds, and was discharged on Day 12 in good condition with scheduled outpatient follow-up.

## Discussion

Mechanical SBO secondary to perforated appendicitis is uncommon in children, with fewer than 50 pediatric cases documented [5]. Adhesion formation from periappendiceal inflammation or abscess often leads to bowel kinks [6]. This rarity contributes to frequent misdiagnosis as gastroenteritis or paralytic ileus, delaying treatment and increasing the risk of perforation and sepsis [3].

Imaging is critical to distinguish uncomplicated gastroenteritis from mechanical obstruction. While plain radiographs can confirm SBO (Figs 1 and 2), they may not reveal its cause. Although CT offers high sensitivity for appendiceal pathology, radiation exposure is a concern in young children. MRI without contrast provides a reliable alternative, demonstrating excellent diagnostic accuracy for pediatric appendicitis [7]. In our case, T2-weighted images identified a periappendiceal fluid collection, mesenteric edema, and an appendicolith (Fig. 3), guiding prompt surgical intervention.

Laparoscopic appendectomy is generally favored for uncomplicated appendicitis, but dense adhesions or perforation may necessitate conversion to open surgery for adequate exposure and sepsis control [8]. In this case, a supraumbilical laparotomy was required due to massive bowel dilation and limited visualization. Intraoperative findings confirmed a perforated appendix with dense phlegmon and adhesions, prompting adhesiolysis, appendectomy, and peritoneal lavage (Fig. 4). Timely surgical intervention and targeted antibiotic therapy typically yield favorable outcomes in pediatric SBO due to appendicitis. Our regimen of meropenem followed by gentamicin for ESBL-producing *E. coli*, combined with early operation, led to rapid CRP decline and an uneventful recovery [9].

Perforated appendicitis presenting as mechanical SBO remains a rare but critical challenge in pediatrics. Prompt imaging-based diagnosis and timely surgical management are essential to prevent severe complications. Close interdisciplinary collaboration among surgeons, radiologists, and pediatric specialists is vital for achieving optimal outcomes in complex appendicitis presentations.

## Data Availability

The data supporting the findings of this study are not publicly available due to the inclusion of confidential patient information. However, they may be made available upon reasonable request from the corresponding author, subject to appropriate institutional review board approval and compliance with relevant regulations.
